# Bypassing Evolution of Bacterial Resistance to Phages: The Example of Hyper-Aggressive Phage 0524phi7-1

**DOI:** 10.3390/ijms26072914

**Published:** 2025-03-23

**Authors:** Maria Rojero, Meagan Weaver-Rosen, Philip Serwer

**Affiliations:** 1 Department of Microbiology, Immunology and Molecular Genetics, UT Health, San Antonio, TX 78229, USA; rojero@livemail.uthscsa.edu; 2 Department of Biochemistry and Structural Biology, UT Health, San Antonio, TX 78229, USA; weaverrosen@uthscsa.edu

**Keywords:** bacteriophage, aggregation of, bacteriophage, hibernation and swimming of, bacteriophage, plaque morphology of, bacteriophage, screening of, electron microscopy, ultracentrifugation

## Abstract

The ideal bacteriophages (phages) for the treatment of bacterial disease (phage therapy) would bypass bacterial evolution to phage resistance. However, this feature (called a hyper-aggression feature) has never been observed to our knowledge. Here, we microbiologically characterize, fractionate, genomically classify, and perform electron microscopy of the newly isolated *Bacillus thuringiensis* phage 0524phi7-1, which we find to have this hyper-aggression feature. Even visible bacterial colonies are cleared. Phage 0524phi7-1 also has three other features classified under hyper-aggression (four-feature-hyper-aggressive phage). (1) Phage 0524phi7-1 forms plaques that, although sometimes beginning as semi-turbid, eventually clear. (2) Clear plaques continue to enlarge for days. No phage-resistant bacteria are detected in cleared zones. (3) Plaques sometimes have smaller satellite plaques, even in gels so concentrated that the implied satellite-generating phage motion is not bacterial host generated. In addition, electron microscopy reveals that phage 0524phi7-1 (1) is a myophage with an isometric, 91 nm-head (diameter) and 210 nm-long contractile tail, and (2) undergoes extensive aggregation, which inhibits typical studies of phage physiology. The genome is linear double-stranded DNA, which, by sequencing, is 157.103 Kb long: family, *Herelleviridae*; genus, *tsarbombavirus*. The data suggest the hypothesis that phage 0524phi7-1 undergoes both swimming and hibernation. Techniques are implied for isolating better phages for phage therapy.

## 1. Introduction

An increase has occurred in lethal, near-lethal [1,2,3,4,5,6,7,8,9], and amputation-triggering [10] infections caused by multi-drug-resistant (MDR) bacteria. Some of the increase was a consequence of hospitalizations during the SARS-Cov-2 pandemic [5]. This increase has revived interest in the use of phages either together with or instead of antibiotics for the treatment of bacterial infections (phage therapy: reviews [11,12,13,14,15,16,17,18,19]). Phage therapy has also had the advantage of no medically significant toxicity [11,12,13,14,15,16,17,18,19], in contrast to the occasional toxicity of antibiotics. The quinolone antibiotics, for example, have been described as being the most actively under development [20] and yet have well-known toxicity, apparently most acutely for the fluoroquinolones [21,22,23]. However, the effectiveness of phage therapy is not yet consistent enough for FDA approval [11,12,13,14,15,16,17,18,19,24].

Previous studies have revealed that the blood lifetime (persistence) of phages is variable by several orders of magnitude [25,26]. The low persistence of some phages appears to be a, if not the, major reason for the observation [11,12,13,14,15,16,17,18,19,24] that phage therapy is not consistently effective. Another source of effectiveness variation is likely to be variability in the anti-bacterial aggressiveness of therapeutic phages, especially for the treatment of biofilms that are not necessarily accessed via blood.

However, at first glance, phages are aggressiveness-limited by self-extinction if they eliminate all accessible bacterial hosts. The self-limiting of aggression and avoidance of this self-extinction is sometimes accomplished by lysogeny, i.e., the combining of the phage genome with a host bacterial genome and the blocking of external phage infection [27,28]. A phage with the capacity for lysogeny (called a lysogenic phage) is counter-indicated for use in phage therapy [11,12,13,14,15,16,17,18,19]. The remaining, more bacteria-destructive (lytic) phages are also aggression-limited, by means that include at least (1) programmed behavior, such as tail-to-tail aggregation [29], and (2) ultimately selection for phage-resistant bacterial mutants [30,31]. The evolution of phage-resistant bacterial mutants is a major limitation of phage therapy [32,33,34].

Apparently not considered in the past, was the possibility that phage therapy could be enhanced by finding and using lytic phages that, at least in the short-term, bypass the evolution of host resistance. This feature will be called a hyper-aggressive feature. The recognition of hyper-aggressive feature-exhibiting phages may have been prevented in the past by the delay in a phage’s manifestation of these features. We were among those who initially disregarded the possibility of phages bypassing the evolution of host resistance. That changed during our work, described here, on the physical, chemical, and biological properties of apparently the most extensively hyper-aggressive phage, 0524phi7-1.

## 2. Results

### 2.1. Hyper-Aggressive Features

**Figure 1:**
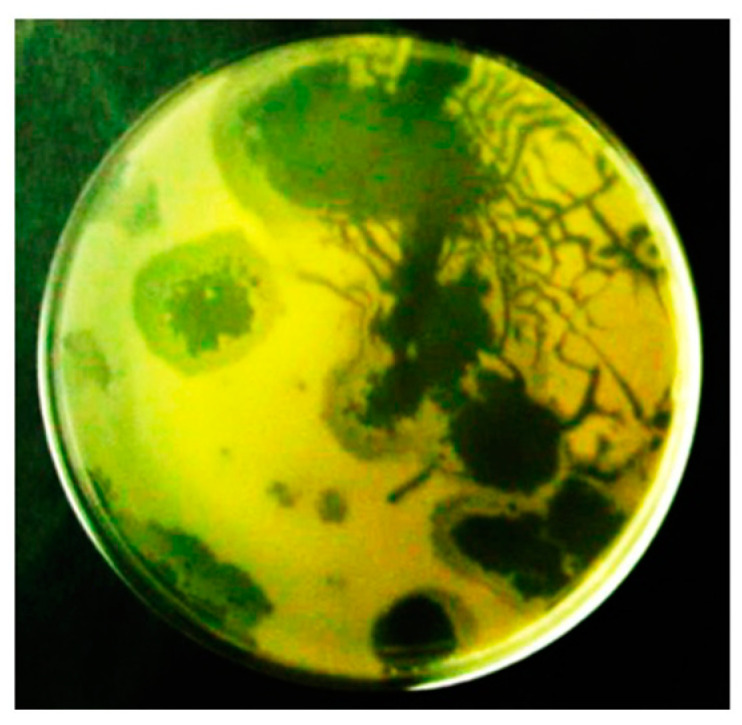
Plating of phage 0524phi7-1 in a 0.2% agarose supporting gel. The plate was incubated for 18.0 h.

**Figure 2:**
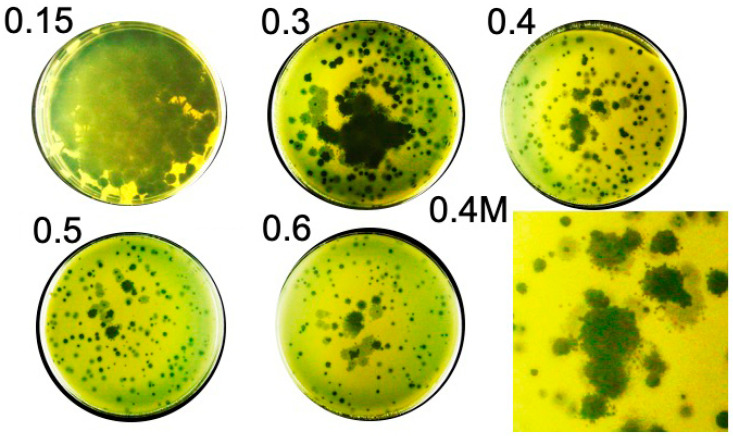
Plating of phage 0524phi7-1 for 18.0 h vs. plaque-supporting agarose gel concentration. The percentage of agarose is indicated at the upper left of the Petri plate. The M indicates a magnified region.

The characterization of phage 0524phi7-1 began during its in-gel isolation from a soil sample that had been obtained, 8 inches deep, from a field used for growing oats. In a 0.2% plaque-supporting agarose gel, this phage generated zones of clearing that were (1) usually asymmetric, (2) often clear at the center, with a surrounding partially turbid region, and (3) often accompanied by clear plaque extensions along narrow channels. This pattern was (1) repeated after single-plaque cloning four times (Figure 1), (2) not caused by gel breakage, and (3) unlike anything previously reported to our knowledge. The previous cloning was twice from the clear regions of 0.2% plaque-supporting gels and twice again from clear, circular plaques in 0.4% plaque-supporting gels where more well-defined plaques were seen, as shown below.

When the concentration of agarose was 0.3% or higher, overnight (18 h) plaques were usually symmetrical, but sometimes not. The symmetrical plaques were (1) sometimes completely clear, (2) sometimes semi-turbid, and (3) sometimes clear in the center with an accompanying, surrounding, semi-turbid region (Figure 2; agarose supporting gel percentage is at the upper left of the panel). One might have proposed the hypothesis that the semi-turbid regions were generated by lysogeny [25,26]. However, as shown below, the semi-turbid regions subsequently cleared, which made this hypothesis inconsistent with the data. In agreement, the genomic sequence (below) has no integrase gene.

Another cause of the semi-turbid regions was suggested by the close observation of some plaques in 0.4–0.6% agarose plaque-supporting gels. Both clear and semi-turbid regions of these plaques were surrounded by smaller, clear satellite plaques (magnified region of 0.4% plaque-supporting gel, labeled 0.4 M, in Figure 2). Thus, the semi-turbid region could also be a region in which (1) phages from the clear region had initiated multiple satellite plaques that were not yet visible, and (2) phages from the satellites were in the process of clearing host cells.

**Figure 3:**
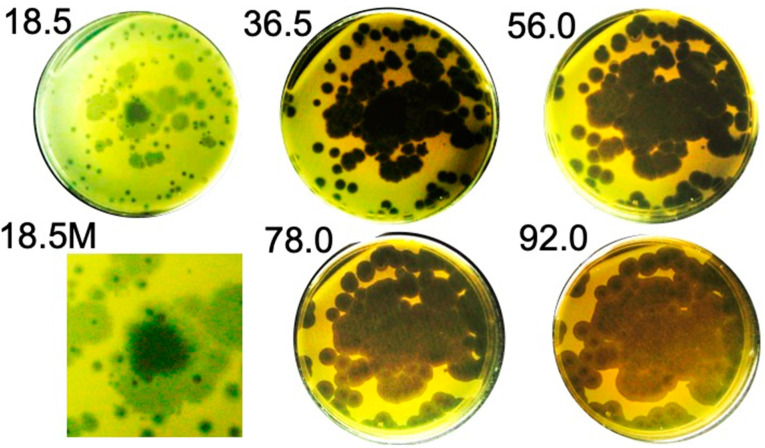
Plating of 0524phi7-1 vs. time in a 0.4% agarose plaque-supporting gel. The time in hour is indicated at the upper left of the Petri plate. The M indicates a magnified region.

In any case, the semi-turbid condition was temporary. Figure 3, in the panel labeled 18.5 at the upper left, shows an 18.5 h incubated Petri plate with a relatively high content of semi-turbid plaques supported by a 0.4% agarose gel. When this Petri plate was incubated for additional time, the semi-turbid regions completely cleared (Figure 3; the time of incubation [hr] is indicated at the top left of the Petri plate).

In addition, (1) plaques, which had become completely clear, continued to enlarge for about 4 days and (2) colonies of phage-resistant bacteria, which typically appear for other phages, did not appear. In the case of coliphage T4, a plating like the one in Figure 3, produced over 300 phage-resistant colonies by 40 h of incubation, the exact number being dependent on the host strain and the number of phages plated. Resistant-mutant colonies (>2000) of a relatively high-resistance-producer host are shown in Supplemental Figure S1. In addition, the *B. thuringiensis* host used here developed permanently phage-resistant cells when the phage was 0105phi7-2. Further evidence of the absence of phage-resistant host cells is in Section 2.4.

Whatever anti-phage defense was present in the host cells, phage 0524phi7-1 bypassed it in time. That is to say, phage 0524phi7-1 exhibited hyper-aggression. As discussed above, phage hyper-aggression is expected to be an asset for phage therapy. Thus, phage 0524phi7-1 was chosen for further characterization.

### 2.2. Fractionation and Genomic DNA

Characterization began with preparative in-gel propagation (plate stock) and partial phage purification by (1) low-extent centrifugation to pellet and remove large cellular fragments, cells, and agarose gel fragments, followed by (2) higher-extent centrifugation to pellet phages and related particles and to remove some more slowly sedimenting (smaller) contaminants. Finally, the latter, pelleted particles were resuspended and fractionated by rate zonal centrifugation in a sucrose gradient.

**Figure 4:**
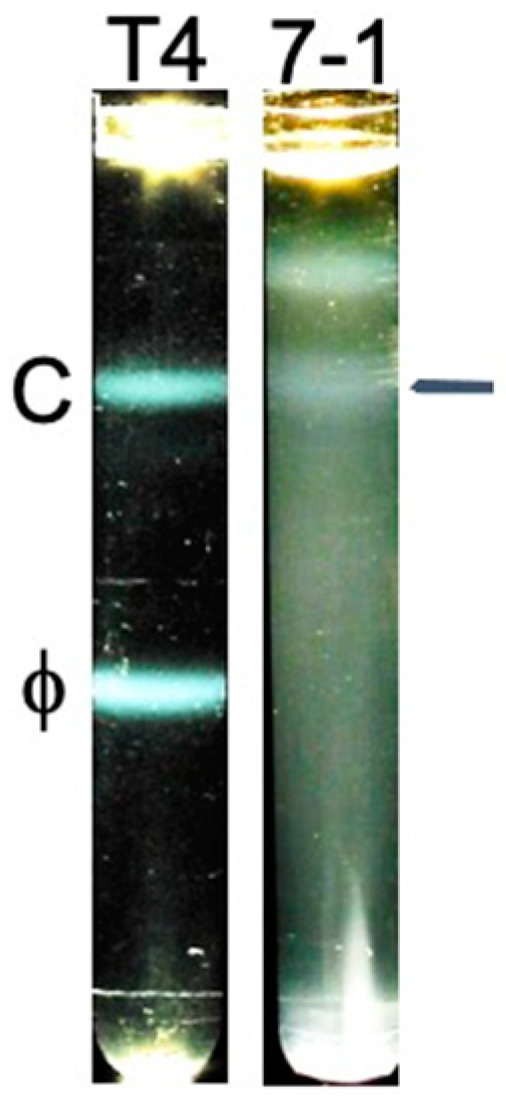
Rate zonal centrifugation in a sucrose gradient of a preparation of phage T4 (centrifuge tube labeled T4) and phage 0524phi7-1 (centrifuge tube labeled 7-1). The image for T4 is reproduced from Ref. [35]. C, capsid; ϕ, phage.

The result (Figure 4, 7-1) was unlike previous results with phage T4 [35] (reproduced in Figure 4, T4) and phage G [36] in the following way. Light scattering for 0524phi7-1 was distributed throughout most of the sucrose gradient, with an approximate distribution of sedimentation coefficients of 200–900. A weak 0524phi7-1 band was present at the lower S values (horizontal line at the right in Figure 4, 7-1); this band was not present in another preparation. In contrast, a much more dominant, symmetrical band of monomeric phage T4 and phage G was observed. The same procedure was used for all three phages.

**Figure 5:**
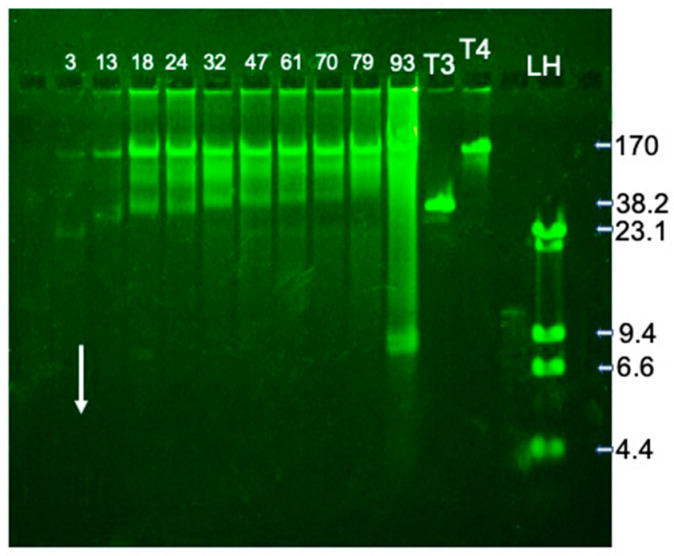
Electrophoresis of the DNA packaged in particles in fractions of the sucrose gradient of Figure 4. DNase-resistant DNA was expelled from capsids and fractionated by agarose gel electrophoresis. The arrow indicates the direction of electrophoresis. Lanes are labeled by the average percentage of the sucrose gradient traversed. The DNA length standards are (1) the DNA of phages T3 (T3) and T4 (T4) and (2) a restriction endonuclease Hind III digest of phage lambda DNA (LH). Phage infectivity titers were the following (lane label, followed by titer ×10^9^ in parentheses): 3, (0.44); 13, (0.52); 18, (10.0); 24, (10.0); 32, (6.4); 47, (4.6); 61, (2.9); 70, (4.3); 79, (3.0); and 93, (4.1).

To locate both phages and phage-related particles in fractions of the sucrose gradient of Figure 4, 7-1, we performed a gel electrophoresis of DNA that was DNase-resistant. Packaged phage DNA, but not host DNA, was expected to be DNase-resistant. After DNase digestion, the sample was exposed to conditions that expelled DNA from phage capsids and removed proteins. Then, the DNA was fractionated by agarose gel electrophoresis. For typical double-stranded DNA phages, expelled genomic DNA has had a unique length and, therefore, has formed a single sharp band during agarose gel electrophoresis (e.g., phage T4 DNA in lane labeled T4 in Figure 5).

For the sucrose gradient of Figure 4, minimal DNA was seen in fractions whose content had traversed 3.0% of the sucrose gradient on average (to be called 3.0% particles; the mean percentage of the sucrose gradient traversed is indicated at the top of a lane in Figure 5). No DNA was observed for more slowly sedimenting particles. For more rapidly sedimenting (13–79%) particles, a sharp band was observed at a position just short of the position of T4 DNA. The implied length is 150 ± 15 Kb. Atypically, the 13–79% particles also had packaged DNA that formed bands that were (1) less sharp and (2) further from the electrophoretic origin, an indication of shorter DNA (Figure 5).

Atypically, none of the bands in Figure 5 had intensities that peaked in any fraction. In addition, the infective particles were, like the packaged DNA, spread throughout the sucrose gradient; plaque-forming units/mlx10^9^ are in the legend to Figure 5.

The co-sedimentation of phages, together with related particles with several different packaged DNA lengths, was also atypical. Typically, the sedimentation rate of a DNA-containing capsid has increased with an increasing length of a DNA molecule packaged within the capsid (see [35,36]). The co-sedimentation of Figure 5 can be explained by aggregation.

### 2.3. Electron Microscopy (EM) and Classification

**Figure 6:**
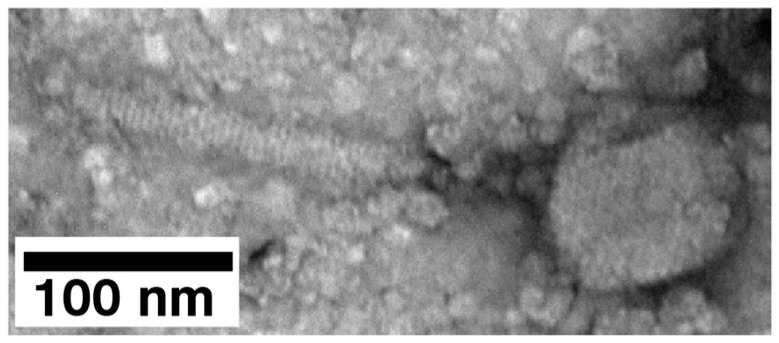
Electron microscopy of a single particle of negatively stained phage 0524phi7-1.

Aggregation was confirmed when electron microscopy was performed with specimen preparation by negative staining with uranyl acetate. Phages and phage capsids in the 24% fraction of Figure 5 were observed to be primarily in aggregates. Deliberate searching did reveal an occasional single phage-like particle (Figure 6). Thus, particle aggregation is, one, possibly the only, cause of the variability in sedimentation rate. Aggregation also lowers the number of PFU observed.

The fraction with these particles also had smaller-than-phage particles, throughout the background (Figure 6). The amount was higher than previously observed with phage 0105phi7-2. Phage 0105phi7-2 had been propagated and processed with a similar procedure. Assuming, therefore, that these smaller particles might have arisen from the degrading of phages and related particles, we tried improving the specimen preparation by using 1.0% sodium phosphotungstate, pH 8.4. However, even more apparent damage was observed when this procedure was used.

**Figure 7:**
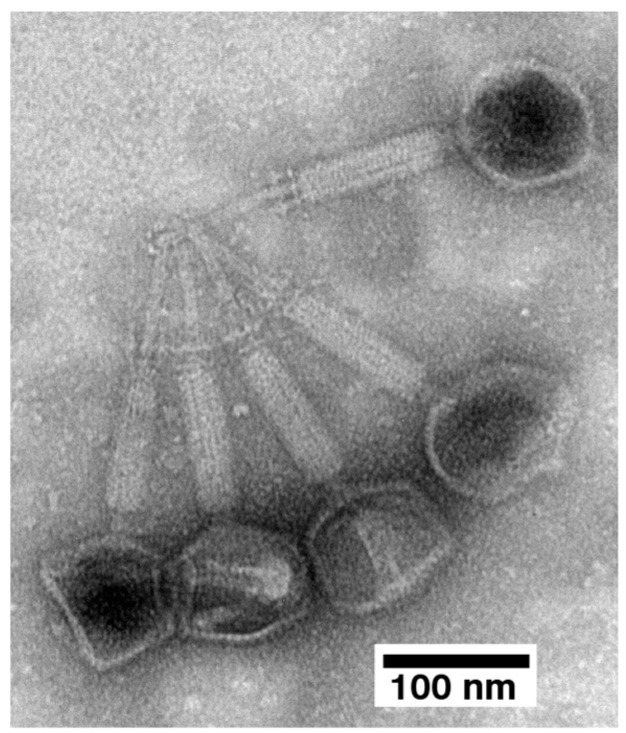
Electron microscopy of aggregated, DNA-emptied capsids of negatively stained phage 0524phi7-1.

The 13% particles from the preparation of Figure 5 were aggregated. They were also more emptied of DNA than the 18% particles observed above. Lower size of the aggregates assisted the imaging of the details. These details included, primarily, the tail–tail aggregation of capsids with emptied heads (Figure 7). The tails were contracted, which classifies 0524phi7-1 as a myophage.

To further test this conclusion, we sequenced the DNA of the 24% particles in Figure 5. The major contig had 157.103 Kb, within the range predicted by the data in Figure 5. The family is *Herelleviridae* (genus: *TsarBombavirus*), members of which are myophages [37]. A more detailed study of the genome is in progress. We note the irony of the genus having been named after the largest hydrogen bomb explosion because 0524phi7-1 appears to be the most host-damaging phage yet isolated (see also Section 2.4, below).

### 2.4. State of In-Gel Propagating Phages: Transfer and Subsequent Propagation

**Figure 8:**
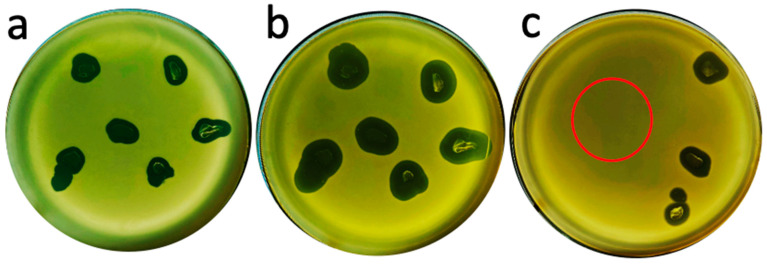
Re-propagation on a mature lawn of in-gel propagating phages: (**a**) 18.0 and (**b**) 40.5 h incubation of six transfers of a plaque to a 1-day-old lawn and (**c**) 18.0 h incubation of three plaque transfers (right) and one 100 PFU liquid transfer (center of red circle).

We next tested the possibility that in-gel propagating phages could be re-propagated on a mature lawn of bacteria, given the potential application to phage therapy. The mature lawn was a model for an extensive surface infection, for example, of skin. We (1) excised pieces of extended plaques in a 0.4% agarose supporting gel, (2) placed them on a 1-day-old bacterial lawn, and (3) re-incubated the Petri plate. A neighboring region of the 1-day-old lawn had cleared after 18 h (Figure 8a) and the cleared region expanded after another 22.5 h (Figure 8b).

However, phage 0524phi7-1 did not propagate on a 1-day-old bacterial lawn when 100 PFU, obtained by dilution in T broth from a sucrose gradient (i.e., phages that were active on fresh bacteria), were inoculated on a 1-day-old lawn and then incubated (Figure 8c; the site of inoculation of 100 PFU is at the center of the red circle). However, dramatic propagation was observed when the upper layer gel from a phage-cleared region was placed on the same mature host lawn and co-incubated in Figure 8c (cleared areas to the right of the red circle). Thus, the infectivity of phage 0524phi7-1 depended on its history.

In preliminary work, we found that one can room-temperature dry the phage-transferring gel and obtain post-drying phage transfer with host clearing. The clearing obtained is, however, less extensive that what is observed in Figure 8. The time of comparable clearing is increased by 1–2 days.

**Figure 9:**
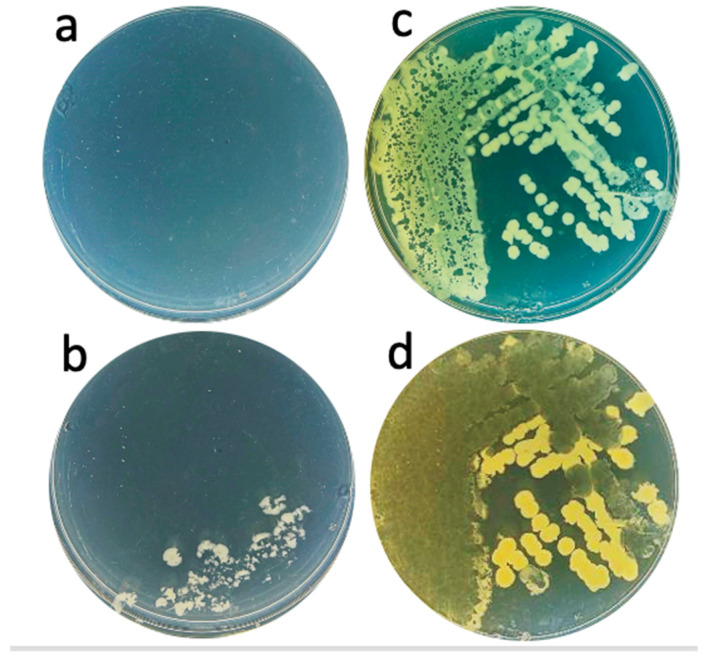
Streaking for bacteria from the following zones: (**a**) clear, 18 h incubation, (**b**) semi-turbid, 18 h incubation, (**c**) turbid, 18 h incubation, and (**d**) turbid, 48 h incubation [of the Petri plate in (**c**)].

We performed the following to further characterize in-gel propagating phages and to determine whether live bacteria were in the phage-cleared region of upper layer agarose gel. An approximately 20 μL sample of gel was streaked on lower layer agar without adding phages. No bacterial colonies were observed after 18 h incubation at 30 °C (Figure 9a).

The same streaking of the contents of a semi-turbid or turbid region, from the same Petri plate, did yield bacterial colonies (semi-turbid: Figure 9b; turbid: Figure 9c). Note that the bacterial colonies in both Figure 9b and 9c had phage plaques within them. A further 30 h incubation enlarged these plaques and reduced bacterial colony-derived turbidity in the region of most dense bacterial growth in Figure 9c (Figure 9d). The region of least dense bacterial growth in Figure 9c was not cleared and the colonies enlarged (Figure 9d). Phages may not have reached this latter region until conditions finally deteriorated to the point that phages could no longer propagate. In any case, the host clearing in Figure 9d is the most extreme example of phage-induced host-clearing of which we are aware.

## 3. Discussion

### 3.1. Improving Phage Therapy for Bacterial Infections

The current data provide context for improving the following two aspects of phage therapy: (1) the aggressiveness of the phages used, and (2) the method used to therapeutically apply the phages. Concerning (1), the (obvious) next step is to attempt the isolation of hyper-aggressive phages for pathogens. The data obtained here suggest that one useful screen is for phages that produce semi-turbid plaques that subsequently clear. In the past, the observation of semi-turbid plaques (before the clearing might have occurred) may have caused such phages to have been incorrectly discarded because of the assumption that the phage was lysogenic. In contrast to semi-turbidity via lysogeny, the semi-turbidity for hyper-aggressive, anti-pathogen phages would be caused by another mechanism, possibly the formation of not-yet-visible satellite plaques. Thus, this screen differs from the current dominant screen for initially clear plaques [11,12,13,14,15,16,17,18,19].

Concerning (2), we begin by assuming that infection-transfer/spreading efficiencies for prospective anti-pathogen, hyper-aggressive phages would be similar to those for phage 0524phi7-1. If so, the phage therapeutic use of in-gel propagating phage transfer, as in Figure 8, would be much more effective than transfer via liquid suspension. Indeed, with in-gel propagating phage transfer, one could test phage therapy potential by a disk-diffusion-like assay (disk-diffusion is the typical assay for the effectiveness of antibiotics [38,39]), as in Figure 8 or, indeed, on the patient’s infected tissue, for example extensively infected skin (in vivo test). 

In contrast to a disk-diffusion assay for antibiotics, with an in vivo test, a successful outcome is also a curative outcome, with potential for deliberate and therapeutic spreading of the curative outcome. The potential for curative spreading would (obviously) be derived from the replicative aspect of phage therapy. Other positive, basic aspects are the (1) absence of detected contaminating bacteria in phage-cleared upper layer gel, and (2) expected trapping by a dried upper layer gel of contaminating large bacterial fragments. These aspects raise the probability that either undried or dried gels, as in Figure 8, can be safely used for the phage treatment of surface infections, without much, if any, further processing. This practice would reduce the cost, time, and complexity associated with making therapeutic phage preparations.

The phage host used here is a potential target for phage therapy. This host bacterial strain is an insect pathogen sometimes used to control insects. A concern exists that the spreading of these bacterial insecticides will cause serious damage to bees. Studies reveal that, although damage to bees does not generally occur with *B. thuringiensis*, under some conditions, it does occur [40,41]. This problem has a potential solution via phage therapy with phage 0524phi7-1.

### 3.2. Analysis of Mechanistic Basics

Phage 0524phi7-1 has four hyper-aggressive characteristics (four-feature-hyper-aggressive phage): (1) the bypassing of the evolution of host resistance, (2) the clearing of semi-turbid plaques, (3) the formation of satellite plaques, and (4) multi-day plaque enlargement. We do not know the extent to which these characteristics are linked to each other. We also do not know the role, if any, that aggregation has in generating phage hyper-aggression.

The formation of semi-turbid plaque regions that subsequently clear and the formation of satellite plaques were explained by the assumption that phages migrated away from the central, clear plaque region, without lysing cells while migrating. Satellite plaques previously seen for *B. thuringiensis* phage 0105phi7-2 [42] behaved as though phage migration was produced by phage attachment to motile host cells while phages were in a non-infective state. The absence of 0105phi7-2 satellites for an agarose plaque-supporting gel percentage greater than 0.2 [42] implied that host cells could not swim through more concentrated gels. Thus, the occurrence of 0524phi7-1 satellite-plaques at agarose percentage as high as 0.6 is evidence that the swimming of phage 0524phi7-1 is the source of the phage motion, given that the host for these two phages is the same.

Swimming is potentially a phage activity evolved to bypass hyper-aggression-induced self-extinction. The rationale is that a phage would swim away from locations that had a high concentration of lysed cells. We do not have sufficient data to propose a signal that would trigger the act and direction of swimming, although the presence of a signal (and evidence for swimming) is suggested by the occasional occurrence of plaque asymmetry. We note that phage T4 has a tail sheath that can act as a GTPase for which a function has not been proposed [43,44]. The function is potentially swimming. Nonetheless, the swimming of phage T4 has not been observed to our knowledge, perhaps because the appropriate propagation conditions have not been used.

Phage hibernation is potentially a second phage activity to bypass hyper-aggression-induced self-extinction. Hibernation would entail a phage’s entering a reversibly non-infective state for a period of time sufficient so that host cells re-enter the location of the phage from another location. The intracellular hibernation-like activity of phage T4 has previously been described [45,46]. The hibernation of phage 0524phi7-1 has already been demonstrated, in that, pre-isolation, 0524phi7-1 was in dry soil for at least 18 years. Before its use for phage isolation, the hydration of this soil sample was too low to support any microbial growth. Furthermore, the depth of the sample (8 inches) implies the possibility that the reversibly non-infective period was greater than 18 years.

### 3.3. The Future

A key question for the future is the following. How is phage 0524phi7-1 achieving its hyper-aggressiveness? Answering this question would add a new component to understanding environmental phage biology and related aspects of phage chemistry and physics. It would also provide information needed to enhance the use of hyper-aggressive phages for phage therapy. This joining of basic science and clinical practice is itself new in that the past basic science-oriented use of phages (to initiate molecular biology, for example) was independent of work on the clinical uses of phages (review [47]).

We are pursuing two types of answers to the above key question: (1) answers based on mechanisms that are independent of any genetic change that occurs during 0524phi7-1 propagation (e.g., the release of compounds that promote infection and/or changes in phage structure), and (2) answers based on mechanisms dependent on genetic changes that occur during 0524phi7-1 propagation. A complete answer may depend on mechanisms of both types. In any case, the hyper-aggressive features of phage 0524phi7-1 appear unique in the current literature.

## 4. Materials and Methods

### 4.1. Isolation and Propagation of Phages

Phage 0524phi7-1 was isolated by in-gel propagation from an 18-year-old, dry soil sample obtained 8-inches deep from a field ploughed for oats (N 27° 30′ 07.3″; W 97° 58′ 19.1″). The initial propagation was in a 0.20% agarose gel (Seakem Gold agarose; Lonza, Rockland, ME, USA) that formed the upper layer above a 1% agar gel (Fischer Scientific, Waltham, MA, USA). Details are in [48]. The growth medium in the lower layer and upper layer gels was 10 g tryptone and 5 g KCl in 1 L of water with 0.001 M sterilized CaCl_2_ added after autoclaving (T broth). The pH of the T broth was adjusted to 6.0 with HCl to improve the propagation of the host and to mimic the pH of the soil sample from which the phage was isolated. The bacterial host was *B. thuringiensis*. Subsequent cloning steps are indicated in Section 2.1. All cloning steps were performed without any liquid suspension of the phage, using procedures previously described [48] and the above medium.

The extensive characterization of the in-gel, plaque-forming propagation of phage 0524phi7-1 (Figure 1, Figure 2, Figure 3, Figure 8 and Figure 9) used the following inoculum: diluted phages from the 24% fraction of Figure 4 and Figure 5.

Phage T3 was propagated in liquid culture by the use of procedures previously described [49]. Phage T4 was propagated in-gel and purified by in-gel procedures previously described [35].

### 4.2. Partial Purification of Phage 0524phi7-1

Phage 0524phi7-1 was partially purified by the use of low-extent centrifugation, followed by high-extent centrifugation. First, preparative propagation was performed in a 0.4% agarose overlay (plate stock) with a phage inoculum of 2000 plaque-forming units (PFU) and the medium used for phage isolation and cloning. The number of Petri plates, 14.5 cm in diameter, was 5. These plates were incubated at 30 ± 0.3 °C for 40 h. Phages were then extracted by (1) removing the upper layer gel with a spatula, (2) adding medium without agarose (2.0 mL per 14.5 cm Petri plate) and then vortexing.

Next, cells, cell fragments, and agarose gel were removed by pelleting via (low-extent) centrifugation at 5000 rpm for 10 min, at 4 °C, in a JLA-16.250 rotor in a Beckman Avanti J-25 centrifuge (maximum g = 4020). The supernatant was collected, and the resulting pellet was re-extracted twice by resuspending in agarose-free medium, followed by re-pelleting.

After the pooling of supernatants, phages and related particles were pelleted by centrifugation at 8000 rpm for 2.0 h, at 5 °C, in a JA-25.50 rotor in a Beckman Avanti J-25 centrifuge (maximum g = 8294). These pelleted, partially purified particles were resuspended in 0.01 P-M buffer: 0.01M Tris-Cl pH 6.0, 0.01M MgSO_4_, and 4% PEG 3350.

Partially purified particles were further fractionated by the use of rate zonal centrifugation in a sucrose gradient. A 10–35% sucrose gradient was pre-poured, above a 0.7 mL, 62% sucrose layer, in a centrifuge tube for the Beckman SW41 rotor. All sucrose solutions were in the following buffer: 0.01 M Tris-Cl, pH 6.0, 0.01 M MgSO_4_, and 6% polyethylene glycol 3350. A sample with a volume of 0.8 mL was layered on top of the sucrose gradient. The sample was centrifuged at 14,500 rpm, at 5 °C, for 80 min in a Beckman SW41 rotor (average g = 38,627). A digital photograph was taken of light scattered from particles in the sucrose gradient. Fractions were collected by pipetting from the top with visual detection of light scattering.

### 4.3. Agarose Gel Electrophoresis of DNA

The following was the method was used to perform an agarose gel electrophoresis of DNA expelled from particles in fractions of a sucrose density gradient. Initially, unpackaged DNA was digested by (1) adding 2 μL of 1.0 mg/mL of DNase I to 18 μL of the fraction and (2) incubating for 1.0 h at 30 °C. The digestion was stopped by adding the following: (1) 3 μL of 0.2 M EDTA, 11 μL of 60% sucrose in 0.9 M Tris/acetate, pH 8.4, and 0.01 M EDTA, and then (2) 1 μL of 35% Sarkosyl and 5 μL of 20mg/mL bromophenol blue. DNA was expelled by incubation in a 75 °C water bath for 15 min. The DNA samples were kept on ice until loaded in a gel for electrophoresis.

To fractionate the expelled DNA, together with linear DNA standards of known length, electrophoresis was performed in a 0.4% Seakem Gold agarose gel. The gel was cast in and submerged beneath the following buffer: 0.09 M Tris-acetate, pH 8.4, and 0.01 M EDTA. Then, samples were layered in sample wells with a 50 μL glass micropipette. Electrophoresis was performed at 0.3 V/cm, 25 ± 3 °C for 20.0 h. Post-electrophoresis staining was performed with a 1/10,000 dilution GelStar (Lonza).

DNA standards used included the following: a Hind III digest of mature phage λ DNA (48.5, 27.5, 9.4, 6.6, 4.4 Kb [50], purchased from New England biolabs, Ipswich, MA, USA), phage T3 DNA (38.2 Kb [51]), and phage T4 DNA (169.903 Kb [52]), the latter two expelled from phages, as described above.

### 4.4. Electron Microscopy

Particles in post-centrifugation fractions of a sucrose gradient were negatively stained for electron microscopy by the use of the following procedure. A sample of a gradient fraction was (1) placed on a carbon support film for 2 min, (2) washed with 5 drops of MilliQ-purified water (MilliQ/Sigma, Burlington, MA, USA), and then (3) washed with 3 drops of either 1.5% uranyl acetate or 1.0% sodium phosphotungstate, pH 8.4, in the same water. The excess stain was removed by wicking with Whatman filter paper #1. The sample was air-dried for at least 15 min.

All specimens were observed in a JEM-1400 electron microscope, operated at 80 kV, in the Department of Pathology, UT Health, San Antonio. Images were recorded with an AMT image capture engine (Version 7).

### 4.5. In-Gel Propagating Phage Transfer and Subsequent Propagation

The following was performed to transfer propagating phage 0524phi7-1 from a plaque to a second, mature bacterial lawn. A phage-free bacterial lawn was generated at 30 °C by procedures used for phage plaques, but without the addition of phages. The time of incubation is in the text. Then, a phage-cleared area of an upper layer, 0.4% agarose gel was excised and placed on the phage-free bacterial lawn. The Petri plate was re-incubated at 30 °C for the additional time indicated in the text.

To test the effect of the drying of the upper layer gel before placing on a bacterial lawn, the upper layer gel was covered with T broth and then dried at room temperature (25 ± 3 °C), in air, on the surface of parafilm (Amcor, Zurich, CH). The dried gel was then placed on the lawn and incubated at 30 °C.

### 4.6. Sequencing and Annotation of the 0524phi7-1 Genome

For sequencing, DNase-resistant DNA from a sucrose gradient was prepared by the procedure in Section 4.3 and was phenol-extracted and then dialyzed against 0.1 M NaCl, 0.001 M Tris-Cl, pH 7.4, 0.001 M EDTA. The DNA was quantified, and a genomic DNA-seq library preparation was made, with an Illumina Nextera XT library preparation kit by following Illumina protocol (Illumina, San Diego, CA, USA). The DNA-seq library was sequenced and analyzed by use of procedures previously described [53]. The GenBank accession number for phage 0524phi7-1 is PV296012.

## Data Availability

With the exception of the sequence of the 0524phi7-1 genome, the data presented in this study are all available within one or more of the following: Figure and Text. The sequence will be presented in a future communication.

## Supplementary Materials

The following supporting information can be downloaded at: https://www.mdpi.com/article/10.3390/ijms26072914/s1.
