# Supplementary material for: Bypassing Evolution of Bacterial Resistance to Phages: The Example of Hyper-Aggressive Phage 0524phi7-1
Source: Int J Mol Sci. 2025 Mar 23;26(7):2914. doi: 10.3390/ijms26072914 (PMC11988461; doi:10.3390/ijms26072914)
Supplement: Supplementary file 1 [file ijms-26-02914-s001.zip › ijms-3491366-supplementary.pdf]

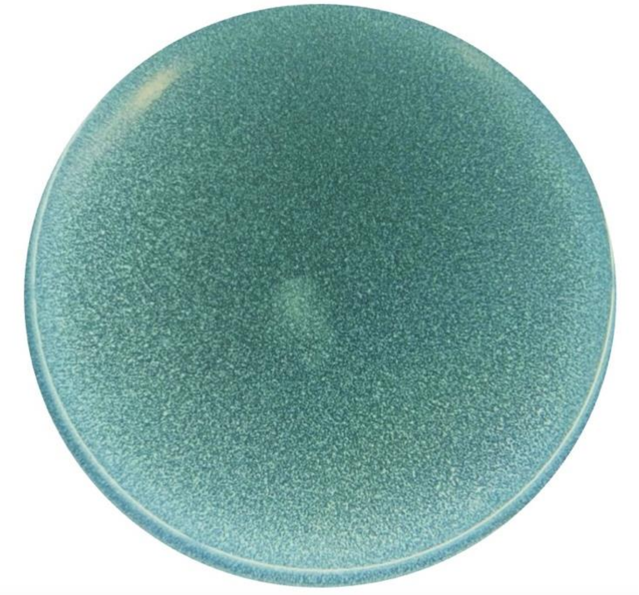

Supplemental Figure 1. Formation, after incubation for 21.0 hr, of *E. coli* colonies resistant to infection by phage T4. The T4 PFU were at near-confluence generating levels in a plaque-supporting gel of 0.5% agar. The host was *E. coli* K-12 HfrC (PO2A) tonA22 garB10 ompF627 (T2-R) relA1 pit-10 spoT1 fadL? phoA4(Am) phoM510 mcrB rrnB2 (λ), a host previously used [25] to plate T4.
